# The Development of Nanoparticles for the Detection and Imaging of Ovarian Cancers

**DOI:** 10.3390/biomedicines9111554

**Published:** 2021-10-28

**Authors:** Edward Henderson, Gabriel Huynh, Kirsty Wilson, Magdalena Plebanski, Simon Corrie

**Affiliations:** 1 Department of Chemical and Biological Engineering, Monash University, Clayton, VIC 3800, Australia; edward.henderson1@monash.edu (E.H.); Gabriel.Huynh@monash.edu (G.H.); 2 School of Health and Biomedical Sciences, RMIT University, Bundoora, VIC 3083, Australia; kirsty.wilson2@rmit.edu.au (K.W.); magdalena.plebanski@rmit.edu.au (M.P.); 3 ARC Training Center for Cell and Tissue Engineering Technologies, Monash University, Clayton, VIC 3800, Australia

**Keywords:** ovarian cancer, nanoparticles, imaging, contrast agents, molecular imaging, intraoperative aids, nanotechnology

## Abstract

Ovarian cancer remains as one of the most lethal gynecological cancers to date, with major challenges associated with screening, diagnosis and treatment of the disease and an urgent need for new technologies that can meet these challenges. Nanomaterials provide new opportunities in diagnosis and therapeutic management of many different types of cancers. In this review, we highlight recent promising developments of nanoparticles designed specifically for the detection or imaging of ovarian cancer that have reached the preclinical stage of development. This includes contrast agents, molecular imaging agents and intraoperative aids that have been designed for integration into standard imaging procedures. While numerous nanoparticle systems have been developed for ovarian cancer detection and imaging, specific design criteria governing nanomaterial targeting, biodistribution and clearance from the peritoneal cavity remain key challenges that need to be overcome before these promising tools can accomplish significant breakthroughs into the clinical setting.

## 1. Introduction

In 2020 alone, there were over 300,000 cases of ovarian cancer and more than 200,000 deaths globally, accounting for approximately 3% of cancer occurrences in women [1,2,3]. It is responsible for almost 5% of cancer fatalities in women and the overall 5-year survival rate for the disease is 47%, which is primarily due to 7 in 10 cases being diagnosed at stage III or stage IV [2,3,4]. As the disease is typically diagnosed after dissemination beyond the ovaries and into the peritoneum, complete resections and treatments are challenging. If detected while the tumor is localised to the primary site (stage I), over 90% of cases will survive 5 years from diagnosis [2]. Unfortunately, currently less than one-third of tumors are diagnosed prior to stage III [2,3]. Following diagnosis and first line treatments typically consisting of cytoreductive surgery and platinum-based chemotherapy, 70% of patients will experience recurrence of the disease [5]. Effective cytoreductive surgery is a critical factor in the treatment of the disease, with a strong association between ‘optimal’ resection (i.e., residual tumor volume ≤ 1 cm) and patient survival times [6,7,8,9]. Some major challenges in ovarian cancer include accurate, rapid diagnosis and characterisation of the disease, maximising tumor resection during cytoreductive surgery and effectively monitoring disease recurrence post-treatment. In addition, the limited effectiveness of screening programs and early detection of ovarian cancer necessitates the need for new tools and technologies for the disease. The recent advances in nanomedicines and nanoparticles for imaging and detection are a promising avenue for the development of effective, clinically relevant tools that may improve patient outcomes in ovarian cancer.

One of the significant reasons why new technologies are critical for ovarian cancer is the limited success of early detection and screening programs for early stage diagnosis. Screening programs aimed at detecting primary tumors have had considerable success across various cancers, such as breast cancer [10,11] or cervical cancer [12]; however, ovarian cancer has seen minimal success and feasibility to date. Large-scale studies on ovarian cancer screening programs to date have demonstrated limited, if any, improvement in survival outcomes [13,14,15,16,17]. In a study of over 200,000 participants as part of the UK Collaborative Trial of Ovarian Cancer Screening (UKCTOCS), annual screening of either ultrasound (US) plus serum levels (“multi-modal” screening), ultrasound alone or no screening was performed. While multi-modal screening allowed for significant advantages in terms of disease stage at detection, this did not translate into an improvement in patient survival [15,17]. However, the more advanced screening methods such as multi-modal screening did reduce the number of false-positive results. It also demonstrated that the value of monitoring over time increases in the concentration of the main diagnostic marker used for ovarian cancer, Cancer Antigen 125 (CA125), relative to baseline levels, rather than using a static threshold system [18,19]. The inability for screening programs to result in effective reductions in mortality despite providing earlier detection indicates issues in translating early stage diagnosis into effective, mortality-reducing treatment options. This highlights the need for technologies that can improve patient outcomes, whether they are tools for the screening and earlier detection of tumors or for the diagnosis, characterisation or treatment of the disease.

Nanoparticle-based nanomedicine is an emerging area of technology that combines nanotechnology and biomedical science to create tools for a variety of applications, such as diagnostics, imaging and therapy. For imaging and detection, nanoparticles may be designed for applications such as contrast enhancement or molecular imaging agents. While contrast agents can be utilized to improve contrast and signal in biological imaging, they have limited mechanisms for on-site retention outside of passive physiological processes and biological interactions, such as vascular flow, and the physico-chemical properties of the particles [20]. Properties such as the geometry and surface chemistry of nanoparticles play a major role in the interactions between the nanoparticles and the surrounding physiological environment, with these “bio-nano interactions” becoming an emerging field in the literature [21,22]. The inclusion of specific affinity components such as antibodies allows for the development of molecular imaging agents that are sensitive to the presence of specific molecules and biomarkers [23]. This allows highly specific contrast to be generated that can provide more in-depth information and classification of the tumor, as well as facilitating higher proportions of the injected dose to be retained on site. 

Over recent years, nanomedicine and nanoparticle sciences have experienced significant developments in the understanding of the complex interactions such materials have with the physiology. Upon injection into humans via various administration routes, such as intravenous or intraperitoneal administration, variations in physicochemical properties such as size, shape and surface coatings can result in significant alterations in particle–cell interactions, biodistribution and clearance pathways [20,24]. As such, the development of robust standards in the design and manufacture of these technologies is essential for replicability and efficacy in both research and in the clinic [22,25]. In recent years, increasing numbers of nanoparticle systems are reaching clinical trials or obtaining regulatory approval, albeit the vast majority applied to therapeutic applications to date [26]. Recent approvals of nanoparticles by the FDA include VYXEOS (2017) or Patisiran (2018), both for therapeutic applications, demonstrated the increasing translatability of nanoparticle systems into clinical products, although engineering challenges such as production upscaling and optimization remain [27,28]. As the science surrounding nanomedicine and nanoparticles continues to develop, it is likely that nanoparticle systems will find a role in the clinic for the detection and imaging of ovarian cancer. 

In this review, we report the recent progress in the application of nanomaterials for ovarian cancer detection, imaging and monitoring, with an aim for identifying the most promising technologies that can be utilized to improve such applications. We classify nanomaterials based on the relevant clinical or pre-clinical imaging modality employed, and in order to maintain focus on translationally relevant materials, we have highlighted only the examples that have shown utility in at least pre-clinical imaging studies, if not in clinical trials. Our definition of nanoparticles covers the size range 1–1000 nm in diameter, with an exception made to include microbubble contrast agents based on their use in ovarian cancer management due to the prevalence of transvaginal and transabdominal ultrasound imaging. We then conclude with some discussion points and future perspectives of nanomedicines in ovarian cancer. 

## 2. Current Approaches for Detecting Ovarian Cancers

The timely and effective diagnosis of ovarian cancer is critical for improving survival rates, with the majority of cases being uncovered at advanced stages with dissemination of tumor mass into the peritoneal cavity and beyond [3]. The time delay between tumor onset and the initiation of diagnosis is due to a lack of specific and alarming symptoms prior to late-stage disease that would otherwise result in potential early diagnoses. Typical symptoms present as abdominal bloating and pain, fatigue and the development of ascites, which is fluid build-up generated after tumor dissemination in the peritoneal region and associated with late-stage tumors [29,30,31]. These symptoms commonly present or reoccur in the months prior to patients seeking clinical treatment, with a study in 2004 by Goff et al. finding that 94% of patients experienced symptoms in the prior year, with 67% of those reoccurring prior to diagnosis [32]. 

**Figure 1:**
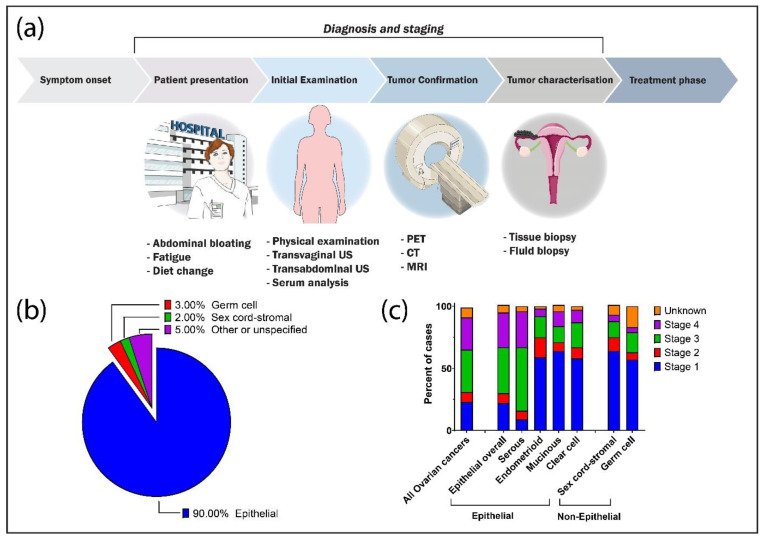
Diagnosis pathway for typical ovarian cancer presentation along with percent occurrence of subtypes and breakdown of diagnosis stage by subtype. (**a**) A schematic flowchart of the diagnostic process from the onset of symptoms to when conventional treatment may begin. After presentation to clinicians, patients may undergo a variety of techniques to confirm and characterise the tumor. In addition, tissue biopsies may be taken simultaneously during cytoreductive surgery, which may then be used for further characterisation. Incidence breakdown by tumor type (**b**) and tumor staging in relation to tumor subtypes (**c**). Data sourced and adapted from Torre et al. [2].

The modern diagnostic pathway, as represented in Figure 1a, involves a combination of imaging, blood tests and surgical biopsy to ascertain the presence of tumors. After presentation of symptoms to clinicians, physical examination of the pelvic region along with transabdominal and transvaginal ultrasounds may be conducted to assess for adnexal masses [29,33]. Critically, these techniques have limited ability to differentiate benign from malignant masses. Subsequent imaging of the region through techniques such as Computed Tomography (CT), Magnetic Resonance Imaging (MRI), Positron Emission Tomography (PET), laparoscopies and colonoscopies can better characterise tumor presence and stage, as well as help differentiate benign and malignant cases [33,34]. Tissue biopsies can finalize confirmation, staging and typing of the tumor, with cytoreductive surgery often being performed in tandem with tissue biopsy. Subsequent pathological confirmation can characterise the disease in terms of histological subtype and stage of the disease, which can then be used to inform further treatment decisions [33].

Depending on disease progression, manual and ultrasound-assisted inspection can lack sensitivity in terms of differentiating benign from malignant masses, especially for smaller and earlier stage tumors [13,16]. Meanwhile, tissue and fluid biopsies are typically performed after symptoms present, often at later stages of disease progression, and risk an invasive procedure for potential false positive cases. The use of biomarkers indicative of ovarian cancer presents opportunities for better pre-operative referral and assessment. As previously mentioned, the current standard in ovarian cancer biomarkers primarily utilize CA125 as it offers good sensitivity at late stages of disease progression; however, it has limited sensitivity in early stage disease (stages I and II) [35,36,37,38]. CA125 levels are elevated in over 80% of ovarian cancer patients; however, this drops to under 60% for stage I disease, limiting its viability for early detection [39]. CA125 also suffers from poor specificity due to it being associated with a number of benign and malignant conditions aside from ovarian cancer, such as endometriosis and various lymphomas [39,40]. CA125 concentrations above approximately 30–35 U/mL are typically associated with an increased risk of malignancy; however, elevation from benign conditions also occurs and can result in a significant false positive rate and misclassification [18,19]. Subsequent attempts to improve this include the Risk of Malignancy Index, or RMI, which utilizes the patient’s ultrasound results, menopausal status and plasma or serum concentrations of the biomarker to differentiate patients into low-risk and high-risk categories [35]. The RMI is reported to improve the diagnostic power of CA125 slightly; however, it still results in large numbers of misclassifications of the disease, despite it being still currently used for diagnostic purposes [37]. This has resulted in the investigation of multi-marker analysis, especially with human epididymis 4 (HE4), and follow-up developments such as the ROMA (Risk Of Malignancy Algorithm) by Moore et al., which utilizes the biomarkers CA125 and HE4 and received FDA 510(k) pre-market approval in 2011 to improve the sensitivity and specificity of biomarker techniques [41,42]. In a 2020 study of 80 women undergoing ovarian removal surgery with stage III-IV high grade serous ovarian carcinoma (HGSOC), Kampan et al. reported sensitivities and specificities of CA125 (96.3%, 84.3%), RMI (96.5%, 86.4%) and ROMA (97%, 91.4%), with improvements in the positive predictive value increasing from 81.8% to 96.7% across the three methods [37]. While biomarker approaches will certainly play a significant role in ovarian cancer diagnostics going forward, this will likely be complementary alongside imaging and biopsy methods for disease staging and analysis. 

## 3. Ovarian Cancer Biology and Microenvironment

Despite the name, ovarian cancer typically refers to tumors originating from a number of different organs in proximity to the ovaries. There are numerous differentiating factors related to ovarian cancer, such as the organ of origin, cellular characteristics or the genomics of the specific tumor, and numerous methods to classify and categorize the disease. Ovarian cancers can be separated into three tumor types depending on their origin, which includes epithelial, germ cell and sex-cord stromal, with cancers of an epithelial origin making up approximately 90% of incidences (Figure 1b) [2,29]. The majority of these epithelial-type tumors originate from the fallopian tube as fimbriae lesions that have subsequently mobilized beyond the fallopian tubes and eventually into the peritoneum. These carcinomas are typically highly aggressive in nature, diagnosed later and subsequently have poorer patient outcomes [43,44]. 

Ovarian cancers of epithelial origins are also conventionally differentiated according to their cellular histology, with the standard subtypes consisting of low-grade and high-grade serous carcinomas (known as LGSOC and HGSOC, respectively), endometrioid, mucinous and clear cell carcinomas [33,45]. Each histological subtype has different molecular profile, risk factors, disease progression and chemosensitivity. As a subtype, serious carcinomas, specifically HGSOC, tend to be diagnosed at late stages of progression (Figure 1c) and make up over 70% of all diagnosed carcinomas as well as making up 70–80% of ovarian cancer deaths [2,46,47]. HGSOC is also associated with mutations in a number of tumor suppression genes, such as TP53, Rb and BRCA1/2 genes in 96%, 67% and 22% of cases, respectively [44,46,48,49,50]. Such mutation profiles significantly differ for non-serous carcinomas [51]. Compared to HGSOC tumors, non-HGSOC tumors are often confined to the ovaries or pelvis, being slower growing subtypes, with over 50% of non-serous carcinomas diagnosed in stage I of the disease [50]. This often enables favourable outcomes for diagnosed patients; however, subtypes such as mucinous and clear cell carcinomas are less responsive to standard chemotherapy. The presence of such tumor heterogeneity further necessitates effective tools for diagnosing and characterising tumors quickly and accurately.

Late-stage HGSOC is also associated with the formation of malignant ascites that often present as symptoms, as mentioned previously. Ascites is caused by the disruption of lymphatic drainage from the region and the production of vasoactive, angiogenic and inflammatory factors in order to suppress the immune response and promote tumor proliferation [52,53]. In a 2017 study, ascites from serous ovarian cancers was shown to increase in vitro cell invasiveness of SKOV-3 cells in human peritoneal mesothelial cells [52] in addition to the significant production of reactive oxygen species when compared to benign and non-serous subtypes of tumors. The authors attributed this to increases in chemotactic agents, reported in previous work [54], as well as potential increases in oxidative stresses on peritoneal mesothelial cells resulting in a breakdown in the barrier function of these cells. Ascites contains numerous tumor and non-tumor related cells and molecules that may be targeted for potential clinical applications, with common examples being vascular endothelial growth factor, interleukin(IL)-6 and tumor necrosis factor (TNF-α), resulting in increasing interest in the analysis and characterisation of the fluid due to its role in late-stage disease [31,37]. 

**Figure 2:**
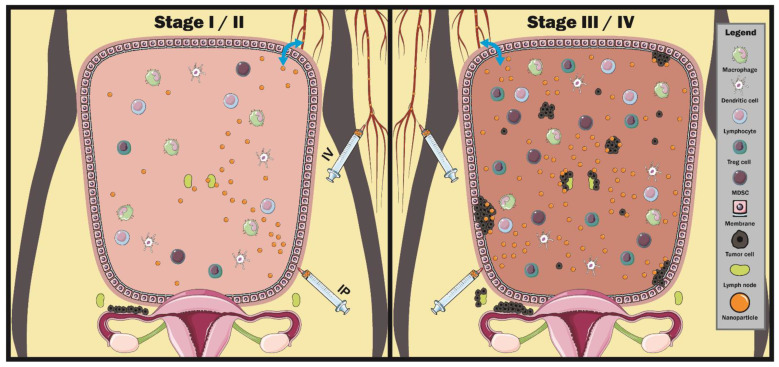
A schematic representation of some key factors and changes from early to late-stage ovarian cancer in terms of cellular microenvironment and nanoparticle delivery. During early stage disease, tumor cells are limited to the ovaries and immediate surroundings and have yet to spread to the peritoneal cavity, with undisrupted peritoneal homeostasis and lymphatic flow through local lymph nodes. Nanoparticles administered intravenous (IV) must circulate through peripheral blood and traverse the peritoneal membrane into the cavity (blue arrows), while intraperitoneal administered nanoparticles (IP) are delivered immediately on-site; however, administered nanoparticles are rapidly cleared via lymphatic flow. During late-stage disease, significant tumor presence in the peritoneal can severely disrupt ascites, resulting in ascites build-up, disruption of lymphatic flow and increased presence of immunosuppressive cells and factors, such as regulatory T-cells (Treg cells) and myeloid derived suppressor cells (MDSCs). This disruption of homeostasis will likely impact nanoparticle biodistribution and clearance from the peritoneal; however, this has yet to be well studied in the literature.

The microenvironment of the peritoneal cavity also contains a variety of cells and signalling molecules that are significantly altered when there is metastatic cancer, such as ovarian cancer. The cavity itself has a multi-layered membrane that lines the visceral, abdominal and pelvic organs and comprises various mesothelial cells and connective tissues that regulate the passage of molecules to and from the cavity [55,56]. Inside, the peritoneum contains vascular and lymphatic networks and associated immunological components such as myeloid and lymphoid-lineage cells such as macrophages, dendritic cells (DC) and lymphocytes (Figure 2) [33]. The presence of tumor metastasis is associated with an increasingly immuno-suppressive environment with greater accumulation of cells such as regulatory T cells (Treg) and myeloid derived suppressor cells (MDSC). There is also an increased presence of chemokines (such as CCL22) and cytokines (such as IL-6, Il-10 and TNF-α) [37,57,58,59] that attract additional immune cells into the cancer environment, as well as cause inflammation. Overall, this environment promotes tumor dissemination within the peritoneal and overall disease progression. 

## 4. Utilizing Nanoparticles in the Ovarian Cancer Microenvironment

The behaviour of administered nanoparticles is dictated by the properties of the nanoparticles as well as the physiological environment in which they are operating. Characteristics such as size and surface charge have well documented effects on in vivo particle behaviour, which has been reviewed previously in the literature [21,22]. Biodistribution and clearance pathways are an example where nanoparticle geometry is critical, with larger sized particles (>10 nm) favouring uptake in the liver and mononuclear phagocyte system, while smaller particles (<10 nm) particles are typically cleared through the kidneys and the renal system [60,61]. The modification of surface chemistry with differing moieties also significantly affects the protein corona that forms on the particles and, subsequently, the interaction with immune components, such as leukocytes and opsonins [24,62,63]. ‘Low fouling’ and ‘stealth’ materials, such as poly (ethylene glycol) (PEG), are standard coating materials that prolong nanoparticle circulation time before clearance occurs. [64]. The inclusion of ‘targeting’ or affinity ligands, such as antibodies or molecules capable of binding specific receptors, is also widespread in nanomedicine in terms of their ability to improve retention of injected doses on site [20]. While nanoparticles will naturally reach target sites by utilizing passive mechanisms such as the circulatory system and regions of enhanced retention, the inclusion of complementary affinity ligands allows preferential retention on specified sites. This can result in an increased percentage of the injected dose reaching the target site, with a 2016 meta-analysis reporting a 50% increase in percentage of injected doses reaching the target across various nanoparticles and tumor models [65]. It can also allow for contrast enhancement of specific molecules, enabling the determination of the presence of such molecules that may be informative to aspects such as tumor type or stage of disease. This makes the inclusion of such ligands ideal for imaging applications, where the enhancement of diagnostically relevant targets without increasing background noise is critical to the technique’s sensitivity and specificity.

Despite ovarian tumors originating in the ovaries and fallopian tubes, significant tumor burden is present in the peritoneal cavity where the disease metastasises, and this environment presents unique challenges for nanoparticle accumulation and clearance. Pre-clinical models of ovarian cancer use a range of tumor models typically consisting of intra-peritoneal or intra-bursal cell implantations (orthotopic models), as well as significant usage of subcutaneous tumor models that are less representative of the native tumor environment. Numerous human derived ovarian cancer cell lines are widely used, such as the SKOV-3 or OVCAR-3 cell lines, while syngeneic mouse cell lines such as ID8 or spontaneous animal models such as in egg-laying hens have also been utilized [66,67]. The peritoneal cavity itself has a multi-layered membranous lining that regulates material diffusion to and from the cavity, as well as a microvascular network to supply required nutrients and remove waste, although only a small fraction of cardiac output is allocated to the peritoneum (Figure 2) [68]. Through the peritoneal membrane, 50–100 mL of fluid subsequently diffuses into the peritoneum and cycles through the lymphatic vessels every hour in healthy women [30,69]. This homeostasis can be disrupted as ovarian tumors develop throughout the peritoneum as metastasis and ascites develop. This has critical implications when considering nanoparticle delivery in ovarian cancer, as nanoparticle biodistribution and efficacy is highly dependent on the interactions between the nanoparticle and its environment [20,70]. Nanoparticles administered in early stages of disease or patients who have not formed metastasises or ascites will likely face high fluid flows and clearance rates that may reduce the time window of imaging or detection [71,72,73]. In contrast, later stages of disease with metastasise and ascites may potentially inhibit nanoparticle clearance mechanisms, which could result in longer imaging times but also toxicity issues; however, this is not well studied in orthotopic tumor models. 

Although the peritoneal membrane is not a significant barrier to small molecules such as low-molecular weight drugs and proteins, there is limited information about the ability of larger particles to diffuse into the peritoneum from peripheral circulation [71]. Coupled with the low proportion of cardiac output going to the peritoneal, IV administered nanoparticles may suffer from low doses reaching the target site, which could result in insufficient signal being generated on-site, although this has not been extensively studied. Alternatively, nanoparticles administered via intraperitoneal (IP) injection, due to the relatively high fluid flow through a healthy peritoneal, are rapidly cleared depending on properties such as size, immune cell interactions and ability to be filtered through the lymphatic stoma [74]. In addition, the visceral peritoneum largely drains via the portal vein, which may likely cause any administered nanoparticles to undergo hepatic metabolism before entering systemic circulation [68]. Clearance rates of nanoparticles following IP administration have been shown to decrease as particle size increases, while single molecules are rapidly cleared within an hour following injection in both healthy and orthotopic tumor bearing mouse models [72,73]. IP administration also presents potential advantages for imaging and detection with nanoparticles, such as higher on-site concentrations while reducing off-site effects, as well as potential longer retention times in the cavity itself [71]. Further investigation of nanoparticles administered by IV or IP will better inform nanoparticle designs when synthesising such materials in the context of ovarian cancer, with such designs being used to optimize the performance of the nanoparticles in their application. 

## 5. Recent Developments of Nanoparticles for Ovarian Cancer Diagnostics

**Figure 3:**
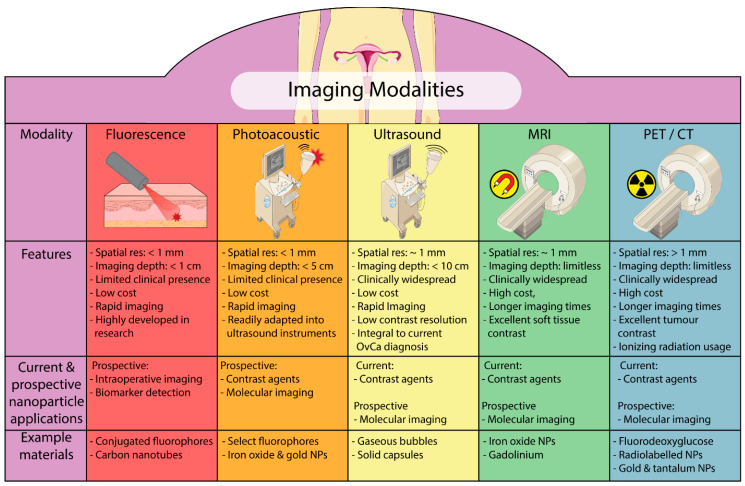
Schematic illustration of the five main modalities of imaging in ovarian cancer: fluorescence, photoacoustics, ultrasound, positron emission tomography/computed tomography and magnetic resonance imaging. In addition, an overview of the features of the modalities and their current and prospective applications related to nanoparticles is also briefly detailed, and some example materials that have been used in the literature are provided.

The use of nanoparticle-based imaging in the context of ovarian cancer diagnostics is under investigation across a broad range of imaging modalities, some of which are already clinically available (e.g., PET, MRI and US) while others are still considered predominantly pre-clinical methods (photoacoustics (PA) and nanoparticle-based optical applications), as outlined in Figure 3. As each modality takes advantage of specific physical phenomena (e.g., photoluminescence, magnetism, radioactivity and echogenicity), the nanomaterials must be synthesised or modified to harness at least one of these mechanisms [75]. For example, materials such as silica are employed across multiple modalities because they are easily synthesised and have highly tuneable parameters (e.g., size, shape, porosity, layers and distinct chemical functionalities) [76]. Such materials typically require additional modification to provide high contrast in any of the imaging modalities, such as by the addition of fluorophores [77]. In contrast, materials such as single-walled carbon nanotubes (SW-CNTs) are inherently photoluminescent and highly photostable, lending themselves to both optical and photoacoustic imaging applications [78,79]. As mentioned earlier, to avoid aggregation and uncontrolled biofouling, nanomaterials require post-modification with anti-fouling polymers such as PEG, although there are also some polymer-based nanomaterials comprising PEGs (or similar anti-fouling polymers) that are rendered anti-fouling based on their synthetic route alone [24,70]. Material selection and subsequent formulation into a nanoparticle requires careful consideration depending on the intended application and whether it is for contrast enhancement or molecular imaging. 

A key aspect when choosing an imaging modality and associated nanomaterial is the imaging depth and resolution required; in the case of ovarian cancer, limited-depth techniques need to show efficacy via trans-vaginal or trans-abdominal routes (e.g., optical, PA and US), while techniques such as PET and MRI do not suffer significantly from penetration depth issues. The imaging resolution required is dependent on the level of detail required and the imaging procedure employed, such as either contrast enhancement, molecular imaging or in vivo biosensing. For imaging, the resolution of the modality will limit the minimum size of a tumor that can be detected, potentially limiting the ability of lower resolution modalities, such as PET, from detecting very early stage tumors [80]. Another issue to consider in terms of depth penetration and image resolution is the type of imaging procedure being performed—namely contrast enhancement or molecular imaging. If molecular imaging is intended, the nanomaterial needs to be capable of binding target cells, molecules or tissues with high-affinity and specificity. Non-specific or interfering signal must be limited and would be a requirement for any imaging process designed to provide molecular classification information to inform treatment options. 

### 5.1. Optical Detection

One of the primary modalities in ovarian cancer, particularly in pre-clinical studies, is fluorescence imaging. This is a case where there are clear delineations between materials that require post-synthesis modification, using molecules such as fluorescent dyes or proteins [23,77], to produce fluorescent signals compared to those that are inherently fluorescent, such as many carbon nanotubes (CNTs) [81,82,83]. The advantages of using optically functional molecules include the wide array of fluorescent materials and the widespread accessibility of fluorescence instrumentation (such as spectrometers, microscopes and purpose-built clinical camera setups [84]). However, the major disadvantage associated with optical detection is high signal attenuation and significant auto-fluorescence in biological environments, severely limiting its functionality for in vivo imaging beyond approximately 1 cm, varying with the tissue’s optical properties [77,84]. While fluorescence is prolific in biomedical research, the clinical use of the modality for imaging is not equally as widespread for this reason. Signal attenuation is reduced at higher wavelengths, subsequently resulting in increased development of near infrared (NIR) wavelength emitters for biomedical and in vivo applications, with the topic well reviewed by Hong et al. [85]. Alongside conventional fluorescence, Raman scattering materials have also seen development for biological applications to overcome high background signal by detecting unique material ‘fingerprint’ spectrums [86]. The advancement of Surface Enhanced Raman scattering (SERS) has resulted in a significant signal generation capacity for the modality and allows Raman-functional molecules and nanoprobes to be detected via unique ‘fluorescent fingerprints’ and distinguished from background signals [87,88]. These advances have resulted in a number of NIR and Raman-functionalised nanoparticles being developed for biomedical imaging and ovarian cancer applications. 

**Figure 4:**
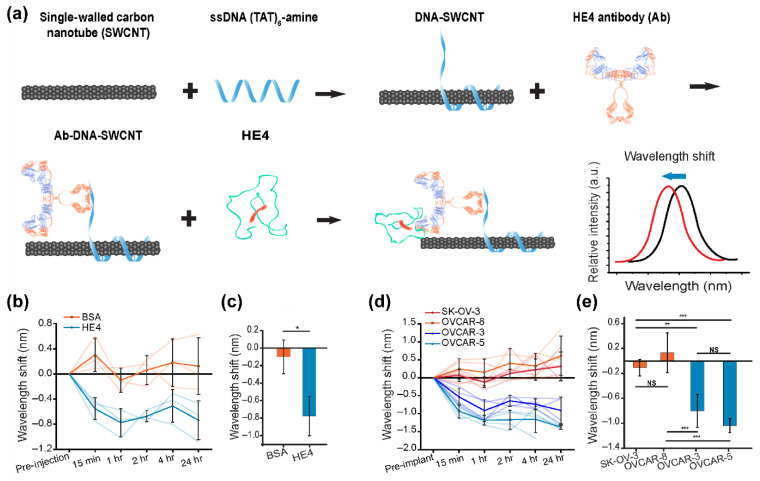
Optically responsive carbon nanotubes sensors sensitive to HE4 biomarker by Williams et al. [83]. (**a**) Single-walled carbon nanotubes were functionalized with DNA and subsequently a HE4 antibody, whereby binding of the complementary antigen would generate a measurable emission wavelength shift. (**b**) Following intraperitoneal implantation of the sensor, emission center wavelength shifts could be detected following IP injections of 10 pmol HE4 and BSA. (**c**) The emission center wavelength shifts 60 min following intraperitoneal HE4 or BSA injected mice compared to control mice (*n* = 3, mean ± SD, * *p* = 0.016, ** *p* < 0.01, *** *p* < 0.001, two-sided *t*-test). (**d**) Variation in sensor emission following implantation in orthotopic tumor bearing mice bearing mice. Sensor showed significant emission blueshift in tumors expressing HE4 (OVCAR-3 and OVCAR-5). (**e**) The emission center wavelength shifts 60 min following sensor implantation in orthotopic tumor bearing mice.

Carbon nanotubes are well suited for biological applications such as molecular imaging and in vivo biosensing due to their inherent optical emission in the NIR-II region [78]. Semiconducting carbon nanotubes have an emission bandgap that can range from 800 nm–1600 nm, which is ideal for optical detection through tissue [78]. In particular, carbon nanotubes have shown excellent applicability in the detection of biological molecules, as biosensors, with a number of designs capable of detecting molecules such as proteins in in vitro and in vivo environments outside of ovarian cancer [82,89]. Specific to ovarian cancer, however, implantable membranes containing anti-HE4 carbon nanotube complexes developed by Williams et al. were shown to function as a variable wavelength probe for HE4 detection (Figure 4) [83]. These nanotubes generate a detectable blueshift in the emission readout after binding of HE4 antigens in in vitro HGSOC fluids (Figure 4b) and distinguished HE4 upregulating from HE4 negative tumors in an orthotopic xenograft model (Figure 4c), where membrane encapsulated nanotubes were implanted across the peritoneal. The presence of HE4 caused a measurable emission blueshift of up to 1 nm, with a detection limit of 10 nM. These implanted sensors were also shown to be effective up to 38 days after initial implantation, and other studies found no significant signs of toxicity on similar carbon nanotubes at periods of up to 4 months, suggesting potential long-term stability of these materials in murine models [90]. Nanotubes of similar design have more recently been used for the probing of a variety of disease-related biomolecules such as DNA [91], RNA [92], protease [93] and proteins [94]. The development of carbon nanotube probes may allow for readily available monitoring of specific biomarkers, which may prove invaluable for detecting ovarian cancer earlier in high-risk groups and in instances of reoccurrence. However, carbon nanotubes are currently limited to in vitro applications, including as sensors, as the field overcomes concerns about toxicity and safety [95,96]. 

The use of fluorescent nanoprobes for intraoperative molecular imaging and guidance in ovarian cancer has been of significant interest, where depth penetration is not a limiting issue. Furthermore, given the effectiveness of cytoreductive surgery on the overall prognosis of disease development [6,7,8,9], allowing surgeons to distinguish between benign and malignant tissue would be highly beneficial. Although a number of studies have demonstrated the utility of fluorescent dyes as intraoperative guides, including in-human studies and clinical trials specific to ovarian cancer (e.g., NCT03180307 and NCT04878094) [97], these studies are typically based around an unconjugated dye system. These dyes are limited by rapid site clearance and photo-bleaching of the fluorophore, limiting the timespan in which they are effective. The addition of a carrier system, such as a dye-loaded nanoparticle, can allow for longer site retention, longer fluorescence lifespan and greater contrast-to-noise, resulting in a more effective guide for tumor debulking [98,99]. Wang and co-workers demonstrated the efficacy of fluorescent nanoprobes for intraoperative imaging and guidance in murine models using peptide-functionalized down-conversion lanthanide nanoparticles that emitted in the NIR-II region [100]. The nanoprobes showed greater signal-to-noise ratio compared to that of clinically approved indocyanine green probes, as well as improved site retention via surface bound epithelial ovarian cancer specific peptides [100]. In a later study by the same authors, a follicle-stimulating hormone peptide was utilized as a binding ligand and showed slightly higher tumor site retention comparatively [101]. Similar recently developed 20 nm polymer nanoparticles reported excellent potential as intraoperative detection of metastatic tumors [102]. The targeted nanoparticles had significant site accumulation immediately after IV injection and were detectable in metastasis up to 36 h post-injection in subcutaneous xenograft ovarian tumors [102]. Given how critical effective cytoreductive surgery is to survival outcomes in ovarian cancer, the development of effective intraoperative tools that can help optimize cytoreductive surgery is a highly promising avenue for development.

Nanoprobes utilizing surface-enhanced Raman scattering have also been recently developed for intraoperative molecular imaging. These nanoprobes developed by Oseledchyk et al. comprised gold nanostars subsequently coated with an organosilica layer with an incorporated Raman reporter and a surface folate receptor for tumor affinity [103,104]. In order to reduce background signal generated by the nanoprobes accumulating on non-specific surfaces rather than the folate receptor target, nanoprobes of a similar design but different reporter and no affinity ligand were used in tandem in a 1:1 ratio. Following intraperitoneal administration of the two nanoprobes in murine models, the folate-targeted nanoprobes coupled to the tumor cells via the folate receptor and were subsequently detectable when using the Raman spectra unique to the nanoprobes, with the non-specific nanoprobes used to reduce non-specific signal of the tumors [103,104]. Nanoprobes of similar design have also been used for intraoperative imaging in other tumor models such as for liver tumors [105] and brain tumors [106,107]. These systems, while utilize a less common optical detection mechanism, are effective at overcoming high background fluorescence, which is a characteristic problem in photoluminescent systems. The development of optical nanoparticles may result in critical advancements in practical ovarian cancer imaging and diagnostics, perhaps most auspiciously as intraoperative imaging agents, which the advantages and limitations of fluorescence are highly suited for. 

### 5.2. Ultrasound

Ultrasound imaging plays a central role in the screening process for ovarian cancer primarily through its use in transvaginal ultrasound, and this is typically performed without the addition of any contrast agents to assist visualisation. The ultrasound transducer generates high-frequency sound waves that are reflected at tissue interfaces and detected in the same device. The detected waves can subsequently be analysed and defined in terms parameters such as amplitude, frequency and wavelength, and the classic B-mode image allows experts to differentiate key anatomical features based primarily on density differences between tissues [108]. Nanomaterials can be designed to provide very high local B-mode contrast, enabling high-resolution imaging particularly of the vascular networks through which the nanomaterials travel, overlaid with anatomical features. Numerous contrast agents, particularly microbubbles, are already clinically utilized for a range of imaging procedures in humans [109], which may ease the pathway of new designs transitioning from research to clinics. 

Given the long clinical history of ultrasound imaging for biomedical applications, including for ovarian cancer diagnosis and monitoring, the modality is widely accessible and highly developed in clinical settings. While ultrasound offers numerous benefits such as low-cost, high throughput and a lack of ionizing radiation, it suffers significantly in terms of its ability to differentiate benign and malignant conditions when compared to alternative methods such as CT and MRI [33]. Performing a transvaginal ultrasound can take under 30 min and does not require large immobile instrumentation to perform. Clinical sensitivities for distinguishing benign from malignant lesions vary from 60% to over 90% across multiple studies, with the UKCTOCS clinical trial reporting a sensitivity of 85% across the 48,000 women who underwent ultrasound screening, while a recent meta-analysis of ultrasound screening for adnexal masses reported a pooled sensitivity of 92% [15,110,111,112]. As mentioned, a critical issue with ultrasound techniques such as a diagnostic or screening tool is its difficulty in differentiating benign from malignant masses, with a poor positive-predictive value of 5.3% in the UKCTOCS trial [111]. The addition of nanoparticles as contrast enhancing or molecular imaging agents could result in the improvement of a critical clinical technique by improving the sensitivity of the test as well as by better distinguishing between ovarian cancer and benign conditions. 

**Figure 5:**
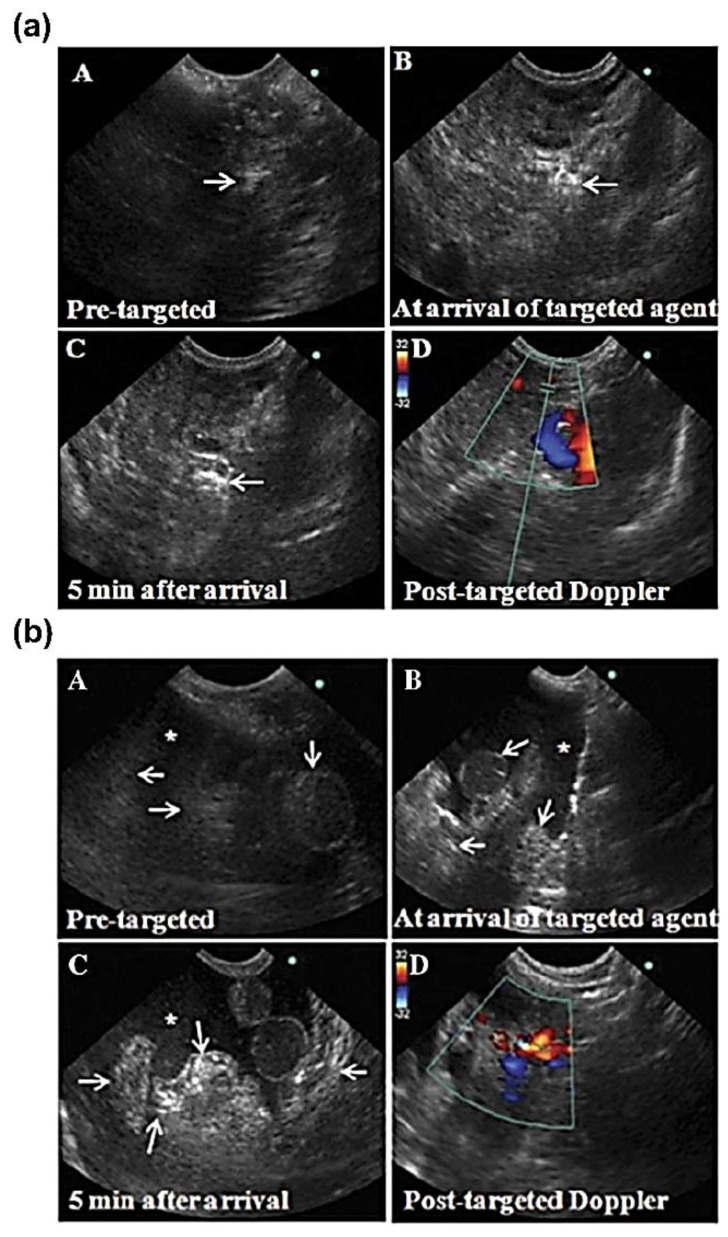
Targeted ultrasound microbubbles in spontaneous ovarian cancer egg-laying hen model by Barua et al. demonstrating the efficacy of relatively simple ultrasound molecular imaging agents in a native ovarian cancer model [114]. Commercially available microbubbles were functionalized with an affinity ligand, death receptor 6 (DR6), and subsequently intravenously administered and imaged at the across multiple time points, with peak intensity generated 5 min after the arrival of microbubbles to the site. The microbubbles significantly increased ultrasound imaging intensities measured via transvaginal ultrasound across early stage ovarian cancer (**a**), where the tumor was limited to the ovary, as well as late-stage disease (**b**). Imaging compared prior to the arrival of the microbubbles (**A**), post arrival (**B**,**C**) and a Doppler scan post-arrival of the microbubbles (**D**). Arrows indicate tumor seeding; * indicates profuse ascites.

Gas-filled microbubbles are widely used as ultrasound contrast agents due to their significantly differing acoustic impedance compared to that of native physiological materials [108]. Microbubbles have short lifetimes once administered, with half-lives under 30 min being typically in vivo. This limits imaging to timeframes in the order of minutes after administration, and the large size required for contrast precludes their penetration from the vasculature into tissues [108,113]. Barua et al. have demonstrated the efficacy of commercially available microbubbles, modified with a variety of tumor-specific affinity ligands expressed on ovarian tumors, in spontaneous tumor models in laying hens, generating tumor-specific contrast up to 15 min post-administration in transvaginal ultrasounds (Figure 5) [67,114,115,116]. These studies demonstrate the efficacy of a relatively simple microbubble-affinity ligand conjugate and that such systems can be detected readily in a natural spontaneous animal model. Similarly, Willmann et al. utilized commercial microbubbles modified with a kinase insert domain receptor, a marker expressed in cancers such as breast and ovarian cancer, as contrast agents in human clinical studies, showing good contrast in imaging up to 30 min post-administration [117]. Although emerging in the field, nanobubbles may be able to overcome the disadvantages associated with the large size of microbubbles in terms of tissue penetration, although studies are currently limited in ovarian cancer applications. Nanobubbles have shown improved permeation into tissue and longer tissue retention times relative to microbubbles in non-ovarian tumor studies [118]. They have also demonstrated good signal accumulation and retention in ovarian tumor bearing mice, however, in recent nanobubbles studies as both untargeted contrast agents and as molecular imaging agents [119,120,121]. Critically, however, these studies are limited to xenograft subcutaneous ovarian tumors, and the technology has yet to be implemented in orthotopic or syngeneic models. Such studies illustrate the promising potential of micro and nanobubbles as contrast enhancing or molecular imaging agents for ultrasound imaging applications.

Solid-state or capsule-like particle systems, which can generate echogenicity from the particle itself or via entrapped echogenic materials, potentially offer better chemical stability and longer retention or circulation times in comparison to microbubbles. This would allow imaging times beyond what gaseous bubble systems can achieve as well as opening up opportunities for additional features, such as drug loading and release. These contrast agents often utilize solid or denser materials relative to biological materials, as in addition gas and liquid-loaded particles, to generate acoustic signal [122]. In addition, multi-interfaced ‘rattle’ particle-in-particle systems offer potentially improved contrast and higher energy utilization over conventional designs [123,124]. A 2019 study by Huang et al. utilized 100 nm nanoparticles consisting of a hybrid polymer-lipid shell with a chemotherapeutic or fluorescent label attached and a perfluorohexane liquid core as an acoustic contrast enhancer [125]. These particles reported comparable performance to that of commercially available microbubbles in a SKOV-3 xenograft mouse model, while remaining stable at the site for at least 24 h post injection [125]. Similar targeted theranostic style nanoparticles have reported relevant accumulation and detectability by ultrasound in xenograft subcutaneous tumor models multiple hours after remote injection in other studies [126,127]. The improved stability and longevity in vivo of these nanoparticle systems over gaseous microbubble systems, allowing for longer time windows in which imaging can be performed, removed the need to re-dose if delays occur between administration and imaging. As a critical modality in ovarian cancer, the development of new technologies for ultrasound for applications such as molecular imaging may be of significant benefit to both imaging and diagnosis and may result in applications with respect to screening the disease.

### 5.3. Photoacoustics

Photoacoustics is an emerging field in biomedicine and cancer diagnostics research and is a modality that integrates the enhanced depth penetration of ultrasound with the high spatial resolution potential found in fluorescence. A combination of both optical and acoustic mechanisms was utilized to generate contrast, whereby the absorption of light by a material causes a localized heating effect, which results in local thermo-elastic expansion and the generation of acoustic waves that can be quantified by an acoustic detector [128]. Photoacoustics allow for sub-millimetre spatial resolutions at significantly greater depths than can be achieved in traditional optical imaging platforms. While endogenous contrast can be generated by physiological components, most notably haemoglobin, the development of contrast agents can significantly improve the relative signal contrast and potentially provide molecular imaging opportunities [129]. Unlike the modalities that are well developed to some extent either clinically or in research, purpose-built photoacoustic instruments are still limited in their availability; however, they can be adapted onto existing ultrasound instruments. In addition, an ultrasound transducer combined with an excitation laser can serve as a makeshift setup if required. These factors make photoacoustics a promising modality for high-resolution imaging that could be deployed alongside ultrasound to generate additional information during imaging procedures. 

**Figure 6:**
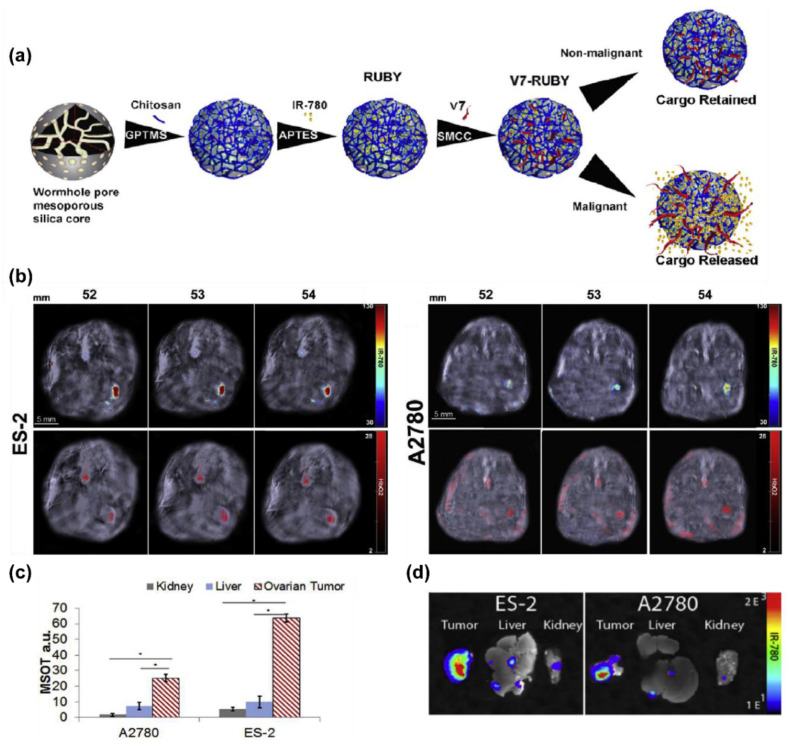
Acidic pH targeted 25 nm mesoporous silica nanoparticles for environmentally sensitive optoacoustic detection and treatment of ovarian cancer developed by Samykutty et al. [133]. (**a**) Schematic representation of the nanoparticle design process. In short, a multistep synthesis procedure generates wormhole-mesoporous silica nanoparticles approximately 40 nm in diameter. A low pH insertion peptide called V7 that, regulated by the chitosan coating, undergoes conformational changes at specific pH conditions (pH = 6.4–6.6), allowing it to intercalate into cell membranes. In these environments, the chitosan protonates, releasing the optoacoustic dye IR780 into the environment, allow for a pH targeted optoacoustic molecular imaging agent. (**b**) Optoacoustic imaging of IR780 (**top**) and oxyhemoglobin (**bottom**) of orthotopic ES-2 and A2780 tumors in athymic mice 4 h following tail vein injection of the aforementioned nanoparticles across selected serial axial slices. (**c**) Biodistribution and accumulation of the nanoparticles indicated a significantly increased presence at the tumor region of interest compared to that of the kidney and liver for both the A2780 (* *p* = 0.00001 and * *p* = 0.00003) and ES-2 (* *p* = 0.0004 and * *p* = 0.00001) models. (**d**) Ex vivo confirmation of the nanoparticles delivery via NIR fluorescence imaging across the tumor, liver and kidneys.

Due to their improved deep tissue imaging relative to optical systems and good contrast generation, nanomaterials with high absorbance and low quantum yields can serve as good photoacoustic contrast agents. Copper sulphide nanodisks and nanoprisms, 25 nm in size, were developed by Wang et al. for in vivo ovarian tumor imaging [130]. Similar nanoprobes have previously reported up to 5 cm of signal penetration depth through tissue [131], as well as demonstrating biocompatibility for in vivo applications [132]. These nanoprobes produced a maximum photoacoustic signal at an optical wavelength of 920 nm in vivo, a good wavelength for depth penetration. The nanodisks subsequently reported a 3-fold acoustic signal over the baseline at 4 h post tail vein administration in an ovarian xenograft mouse model [130]. In another study, specialised mesoporous silica nanoparticles were designed to release an infrared and optoacoustic dye, IR780, into the local environment when the environmental pH range is 6.6–6.8, while remaining entrapped within the nanoprobes outside of this pH range (Figure 6a) [133]. Four hours after tail vein administration on orthotopic xenograft mice, these nanoprobes reported significant signal response from the slightly acidic tumor site (Figure 6b–d) compared to the liver and kidneys following multispectral optoacoustic tomographic imaging, while unbound IR780 dye had no detectable signal. A number of typically high-wavelength optical dyes, such as IR780, and some cyanine dyes generate substantial photoacoustic signal due to a low optical quantum yield, enabling simple multi-modal systems to be synthesised [134]. Multi-modal photoacoustic nanoparticles can also be generated with the addition of photoacoustic dyes, such as indocyanine-green, to ultrasound particles, with such systems having been utilized for photoacoustic-optical imaging applications in ovarian xenograft models [135,136].

Iron oxide and gold nanoparticle systems have shown an excellent propensity for photoacoustic nanoprobes, producing detectable acoustic signal from a range of optical excitation wavelengths, including in the NIR spectrum. These materials are also widespread in research due to their good biocompatibility and varying functionalities and are well developed as commercially available products for biomedical usage [137,138]. A number of Iron Oxide Nanoprobes (IONPs) have been developed as photoacoustic molecular imaging agents, and they target human epidermal growth factor receptor 2 (HER2), which is a protein overexpressed in various ovarian cancer subtypes [139,140]. IONPs functionalized with anti-HER-2 fragment antibodies and a NIR dye have been demonstrated to be able to be detectable in orthotopic HER-2 positive ovarian tumors at 10 mm depth via photoacoustic detection [141,142]. Similar nanoparticles have more recently shown efficacy as duel-PA MRI molecular imaging agents in gastric and pancreatic cancer cells in vivo, showing excellent promise that such systems could find a use across various tumor lines [143]. Alternatively, theranostic gold nanoparticles coated with reduced graphene oxide recently reported NIR-II photoacoustic wavelength imaging capacity and were able to generate significant contrast with minimal background in an ovarian xenograft model and, subsequently, were able to treat the tumor with photothermal therapy [144]. The full application of photoacoustic nanoparticles has yet to be realised, including for ovarian cancer; however, the potential for high-resolution imaging at depths greater than what can be achieved by optical reporters may be invaluable to the detection of small tumor masses and can be utilized in a complementary role to ultrasound detection methods.

### 5.4. Magnetic Resonance Imaging

The use of MRI is widespread in biomedical applications due to the excellent soft tissue contrast it generates, alongside the use of non-ionising radiation and negligible depth penetration limits. MRI is already utilized as an effective tool in the detection and imaging of ovarian cancer without exogenous assistance, with sensitivities and specificities for detection reported at approximately 90% and 80%, respectively, for ovarian tumors [34,110]. Magnetically functional nanoparticles can potentially improve an already powerful diagnostic tool by providing further contrast enhancement to general anatomical features or to specific molecules of interest. MRI contrast agents affect the longitudinal or transverse relaxation times of surrounding material, such as water and tissue, which are known as the T_1_ and T_2_ relaxation times, respectively [145]. Clinically, contrast agents that affect the T_1_ relaxation times appear as regions of bright ‘positive’ contrast during imaging, while T_2_ relaxation contrast appears as darker ‘negative’ contrast. This can limit the interpretive power of T_2_ contrast agents in many cases as a lack of background signal, such as in the peritoneal cavity, renders negative contrast systems difficult to visualise. 

**Figure 7:**
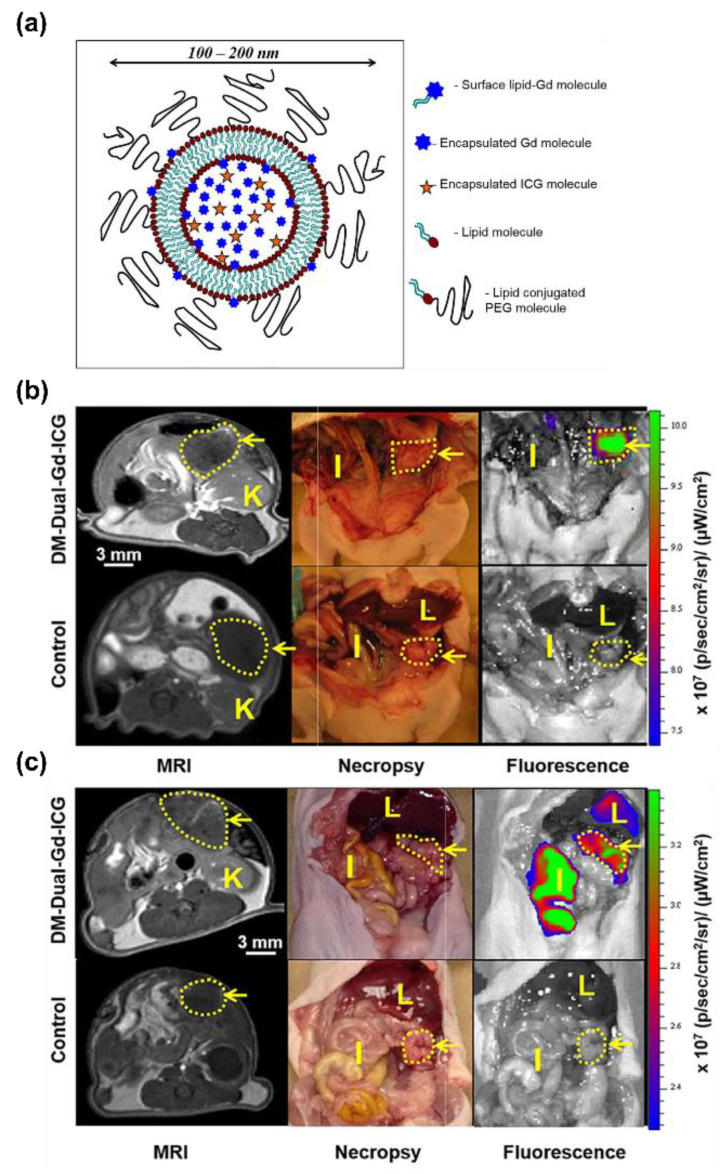
Dual-mode dual-gadolinium-ICG < 200 nm liposomal nanoparticles for MRI and NIR imaging of orthotopic ovarian tumors by Ravoori et al. [146]. (**a**) Schematic diagram of the dual-Gd ICG liposomes consisting of encapsulated Gd and ICG in a lipid bilayer with additional surface bound Gd molecules and a low-fouling PEG molecule. Representative axial MRI (live) and optical (coronal necropsy) imaging of nude mice 2 days post IV injection of the liposomal contrast agent for mice bearing orthotopic OVCAR-3 (**b**) and HeyA8 (**c**) tumors. Arrow, tumor; I, intestine; K, Kidney; L, Liver.

A wide array of materials can be utilized in order to develop magnetically sensitive nanoparticles with differing magnetic properties and physiological interactions. Gadolinium based contrast agents are the current standard in clinical MRI contrast agents as a T_1_ contrast agent generated by the large number of unpaired electrons and its paramagnetism at physiological conditions. The integration of gadolinium into nanoparticle structures allows for the simple synthesis of nanoparticle-based MRI contrast agents. Liposomal constructs with bound and encapsulated gadolinium and indocyanine green (ICG) (Figure 7a), synthesised by Ravoori et al., reported significant detectable signal via MRI on nude mice bearing SKOV3 intraperitoneal tumors following intravenous injection (Figure 7b,c), which was confirmed with follow-up fluorescent imaging [146]. Similar liposomal systems containing gadolinium have been employed in MRI as theranostic nanoparticles in ovarian non-peritoneal tumors, utilizing the liposomes capacity as a vehicle for loading both contrast materials and therapeutic components [147,148]. In addition to liposomes, targeted theranostic nanoemulsions containing gadolinium and chemotherapeutics have been developed and report significant contrast generation both in vitro and in subcutaneous xenograft SKOV3 tumors up to 24 h post-administration [149,150]. These targeted emulsions were slower in terms of reaching comparable levels of contrast than commercially available non-specific contrast agents depending on the affinity ligand bound but demonstrated significantly prolonged contrast generation in comparison. The ability to load gadolinium into nanoparticles, alongside its T_1_ contrast generation, is promising for the development MRI-functional deep tissue contrast and molecular imaging agents for ovarian cancer applications. 

Iron oxide, such as in the form of ferromagnetic Fe_2_O_3_ or ferrimagnetic Fe_3_O_4_, has a variety of uses in medicine due to their magnetic properties, biocompatibility and numerous synthesis methodologies. This includes the generation of MRI contrast-enhancing particles, and several reviews are available describing this topic [151,152]. These materials can function as either T_1_ or T_2_ contrast agents depending on their size, with nanoparticles in the size range of only a few nanometers reportedly acting as a T_1_ contrast agents while larger particles typically alter T_2_ relaxation times [153,154]. Typical designs utilize an iron oxide core subsequently coated with materials such as polymers and affinity ligands for chemical stability, biocompatibility and improved tumor retention [142,155,156,157]. Multifunctional 15 nm iron oxide MRI contrast agents functionalized with an anti-fouling polymer, HER-2 affinity ligand and a NIR-I fluorescent dye reported by Satpathy et al. demonstrated detectability across both MRI and fluorescence of the particles in an orthotopic ovarian tumor model [142,155]. The authors noted in both studies, however, that the T_2_ ‘negative’ contrast generated by the particles has limited uses in applications where significant regions of low background signal is present, such as in the peritoneal cavity, and that T_1_ contrast enhancement would be ideal. What is also noteworthy in these studies is that these modified particles were also able to function as optical and photoacoustic contrast agents, which are properties that were also investigated in other studies [141,142,155]. In a different study, 7 nm iron oxide nanoparticles were formed into 92 nm clusters and coated with Chlorin e6, a clinically utilized photosensitizing agent, and demonstrated efficacy as an MRI theranostic contrast agent [158]. These nanoclusters generated significant T_2_ contrast and were detectable up to at least 24 h post-IV administration in subcutaneous ovarian tumors in nude mice and were able to slow tumor growth via photothermal therapy. Due to the inherent magnetic and photoacoustic properties of various iron oxides, it is expected that these materials will continue to be of significant interest for nanoparticle designs across the modalities of MRI and PA in ovarian cancer.

### 5.5. Positron Emission and Computed Tomography

Positron emission tomography (PET) and computed tomography (CT) are radiological imaging techniques widely used in biomedical imaging. PET imaging relies on exogenous positron-emitting radioisotopes to generate contrast in vivo, with radiotracers such as fluorodeoxyglucose (FDG) utilized across oncological imaging. While this requires the administration of an exogenous component for imaging, background signal is negligible, making PET sensitive to nano or picomolar concentrations of contrast agents [159]. CT utilizes X-ray imaging and typically use endogenous contrast from physiological components; however, soft tissue contrast is highly limited due to X-ray attenuation rates being similar between these materials. Due to their negligible signal attenuation through tissue, PET and CT excel at deep tissue imaging applications, making them well situated for imaging ovarian cancer and the peritoneal cavity. In addition, imaging capabilities such as CT or MRI are often integrated into PET instruments to generate multi-modal imaging systems. This improves the information obtainable from the imaging, allowing for better distinction between anatomical features, with these systems likely to become increasingly common as the modalities continue to develop [160,161].

There are limited numbers of studies that specifically use nanoparticle-based systems for PET/CT imaging in ovarian cancer. This is likely due to the significant presence of ultra-small molecule radiotracers, such as FDG, that are highly effective in PET, while CT typically relies on endogenous contrast or radiographic dyes, such as iodine. In addition, due to the ionizing radiation of PET radiotracers, typical advantages for nanoparticles such as increased in vivo circulation and retention times are less favourable. Time taken to incorporate the radiotracer into the nanoparticle is also important due to the radioactive decay of the tracer and limits how effective longer in vivo lifespans can be. This half-life for biomedical radiotracers typically ranges from minutes, with FDG having a half-life of 110 min, to a few days, such as the longer-lived zirconium-89 (78.4 h) [162]. FDG has high uptake in metabolically active regions, such as tumors, resulting in a highly effective contrast agent and feature as the primary contrast agent across numerous clinical trials (e.g., NCT03811899, NCT04504110 and NCT03604315), and there are numerous reviews on the role of FDG-PET in ovarian cancer [161,163,164,165]. In this respect, simpler radiotracer-conjugate systems are much more widespread throughout the field, with much less driving force to incorporate these systems into nanoparticles compared to what may be found in other modalities [166,167]. 

Outside of the radiolabel-conjugate based contrast agents, there are a few examples of recently developed particle-based PET and CT contrast agents for ovarian cancer. Nanoparticles devised of lipoproteins bound with copper-64, 20 nm in size, have previously been shown as effective PET contrast agents for orthotopic SKOV3 tumors in mice, with the vehicular capacity for loading other molecules such as fluorescent labels or drugs [168]. Additionally, folate-receptor targeting nanoparticles in the form of self-assembling biopolymer (100 nm) and dendrimer (5 nm) nanoparticles have been recently developed for ovarian cancer applications; however, they have yet to be studied in orthotopic tumor models [169,170]. Similarly for CT, nanoparticles devised of tantalum, mesoporous silica and surface attached gold nanoparticles, 166 nm in size, have demonstrated excellent CT contrast in a peritoneal tumor after intraperitoneal injection [171]. The encapsulation of perfluorooctyl bromide (an existing CT contrast agent) in targeted polymers nanoparticles has also been shown to be a potential CT molecular imaging agent in ovarian xenograft subcutaneous models [172]. Additional X-ray contrast materials, such as gold or tantalum, have also seen development in biomedicine; however, they have not yet been applied in ovarian cancer applications [173]. While this is only a limited number, these systems highlight the viability of nanoparticle systems in PET and CT imaging and some of the advantages it can present, such as loading capacities and better control over residence times. However, because PET has an extremely high sensitivity and due to the use of radiolabels, larger loadings of contrast agents and better retention are not necessary and may be detrimental to patient health.

## 6. Conclusions and Future Perspectives

In summary, there are a wide variety of nanoparticles across the various related detection modalities being developed for applications relating to ovarian cancer diagnostics and imaging. In line with current clinical imaging procedures that employ ultrasound approaches, the continual improvement of microbubble platforms for both contrast enhancement and molecular imaging for clinical applications appears to be a promising line of enquiry. Given the importance of cytoreductive surgery and surgical biopsies, there is also an opportunity for intraoperative fluorescence imaging to help distinguish between tumor and non-tumor tissue. The emergence of multi-modal nanoparticles is also a common feature in the literature; while applications for multi-modal nanoparticles imaging/therapy procedures may still be many years away, a more immediate application may be in aiding translation of nanoparticle technologies from pre-clinical trials (e.g., widespread presence of fluorescence and optics) into clinical products (e.g., hospital-based MRI, PET and ultrasound). Given the sobering recent reports that population-based screening approaches are not yet able to affect survival rates significantly [17], the development of safe and effective imaging approaches for the detection of primary tumors may be a judicious direction of attention. 

One of the primary design factors to consider when utilizing nanoparticle technologies in ovarian cancer is the nature of the tumor and its development, with tumor occurrence primarily located in the pelvic and peritoneal regions. Although there are numerous studies of nanoparticle systems for ovarian cancer applications, the vast body of the literature is based on subcutaneous xenograft tumors in immunocompromised murine models. In comparison, only a limited number of in vivo studies have been performed in orthotopic tumors or syngeneic models. This removes both the native tumor environment, a critical aspect relating to nanoparticle biodistribution and clearance, as well as the bio-nano and immune interactions that occur between the physiology and biomedicines. Numerous syngeneic models for murine ovarian cancer have been developed, such as the ID8 [66,174], MOSE [175] and STOSE [176] models, which would serve as more representative models. The inclusion of such models would better capture the full scope of the complex interactions occurring in the biology alongside the interactions between the biology and administered nanoparticles or nanotechnology. The use of models such as these would certainly alter aspects such as the proportions of administered nanoparticle dose reaching the tumor region, their subsequent interactions with the tumor environment and tumor itself and their subsequent clearance from the body [24,63]. Given how critical these factors are when nanoparticles are considered for a clinical setting, especially where longer nanoparticle residence time is desirable such as in imaging and detection of nanoparticles, syngeneic models could better assess the performance of such materials compared to xenograft or non-orthotopic tumor models. 

Detection and imaging are important factors in the diagnosis and staging of ovarian cancer, with a number of developments in nanomedical molecular imaging and contrast agents. Nanoparticles that are functional in at least one detection modality can be found across the varying modes of detection, particularly fluorescence, ultrasound and magnetic resonance imaging. However, developments have yet to significantly transition from lab-based cell cultures and xenograft subcutaneous murine models to more clinically deployable systems. Further development on the bio-nano interactions of nanoparticles in ovarian cancer microenvironments is also required if designs are to be optimized, in addition to considerations around reproducibility and scale-up of production. The inclusion of syngeneic models in the developmental phase will also help bridge the transition from pre-clinical to clinical technologies. This would help drive the development of clinically functional tools that can empower clinicians and improve survival outcomes for patients.
